# Refractoriness of *Sergentomyia schwetzi* to *Leishmania* spp. is mediated by the peritrophic matrix

**DOI:** 10.1371/journal.pntd.0006382

**Published:** 2018-04-04

**Authors:** Jovana Sadlova, Miroslav Homola, Jitka Myskova, Magdalena Jancarova, Petr Volf

**Affiliations:** Department of Parasitology, Faculty of Science, Charles University, Prague, Czech Republic; Instituto Oswaldo Cruz, BRAZIL

## Abstract

**Background:**

The peritrophic matrix (PM) is an acellular chitin-containing envelope which in most blood sucking insects encloses the ingested blood meal and protects the midgut epithelium. Type I PM present in sand flies and other blood sucking batch feeders is secreted around the meal by the entire midgut in response to feeding. Here we tested the hypothesis that in *Sergentomyia schwetzi* the PM creates a physical barrier that prevents escape of *Leishmania* parasites from the endoperitrophic space.

**Methodology/Principal findings:**

Morphology and ultrastructure of the PM as well the production of endogenous chitinase in *S*. *schwetzi* were compared with three sand fly species, which are natural vectors of *Leishmania*. Long persistence of the PM in *S*. *schwetzi* was not accompanied by different morphology or decreased production of chitinase. To confirm the role of the PM in refractoriness of *S*. *schwetzi* to *Leishmania* parasites, culture supernatant from the fungus *Beauveria bassiana* containing chitinase was added to the infective bloodmeal to disintegrate the PM artificially. In females treated with *B. bassiana* culture supernatants the PM was weakened and permeable, lacking multilayered inner structure; *Leishmania* colonized the midgut and the stomodeal valve and produced metacyclic forms. In control females *Leishmania* infections were lost during defecation.

**Conclusions/Significance:**

Persistence of the PM till defecation of the bloodmeal represents an important factor responsible for refractoriness of *S*. *schwetzi* to *Leishmania* development. *Leishmania major* as well as *L*. *donovani* promastigotes survived defecation and developed late-stage infections only in females with PM disintegrated artificially by *B*. *bassiana* culture supernatants containing exogenous chitinase.

## Author summary

Phlebotomine sand flies are the main vectors of *Leishmania* parasites. However, only about ten percent of the described sand fly species are proven or suspected vectors. Several factors controlling vector competence act during the early phase of infection preceding defecation of bloodmeal remnants. Sand flies of the genus *Sergentomyia* including *S*. *schwetzi* were repeatedly suggested to be involved in *Leishmania* transmission in Africa. Here, we tested the hypothesis that *S*. *schwetzi* is refractory to all *Leishmania* species tested due to the long persistence of the peritrophic matrix, the chitinous envelope which surrounds ingested blood within the sand fly midgut. Addition of exogenous chitinase to the *S*. *schwetzi* infectious bloodmeal led to disintegration of the peritrophic matrix which allowed *Leishmania* parasites to escape into the midgut and produce mature infections with colonization of the stomodeal valve and generation of infective metacyclic forms. Parasites in control flies were not able to escape from the peritrophic matrix and were lost with the defecation of blood remnants. The study strongly suggests that in *S*. *schwetzi* the peritrophic matrix forms an important barrier for the development of *Leishmania* parasites.

## Introduction

Sand flies (Diptera: Psychodidae) are the vectors of *Leishmania* species (Kinetoplastida: Trypanosomatidae) parasitizing humans. Over 800 species of sand flies have been described to date but only 98 species are proven or suspected vectors of human leishmaniases [1,2]. Development of *Leishmania* infections in the sand fly vectors is a complex, often species-specific process (reviewed by [3–5]). Some sand fly species can be infected with various *Leishmania* species (permissive vectors); other species are considered specific (restrictive) vectors which are only able to harbour the *Leishmania* species that they transmit in nature (e.g. *Phlebotomus papatasi* and *L*. *major/L*. *turanica)* [4,6,7].

Factors controlling vector competence act during the early phase of infection preceding or accompanying the defecation of the bloodmeal remnants. Blood digestion and early development of parasites occur inside the peritrophic matrix (PM), an acellular chitin-containing envelope which protects the midgut epithelium against damage and compartmentalizes the midgut into endo- and ectoperitrophic spaces [8]; in some hematophagous insects the PM performs also a central role in heme detoxification [9]. In sand flies, the PM was suggested to protect parasites against the action of digestive enzymes during the early hours post blood feeding [10]. Inside the endoperitrophic space surrounded by the PM, *Leishmania* amastigotes ingested with the bloodmeal transform to procyclic promastigotes. They must overcome the activity of the digestive enzymes, replicate and then escape to the ectoperitrophic space. This escape coincides with the transformation of procyclic forms to elongated nectomonads [11] which attach to the midgut epithelium to avoid expulsion from the midgut during defecation of undigested blood remnants. Failure of either (i) resistance to harmful environment caused by blood digestion, (ii) escape from the endoperitrophic space or (iii) attachment to the midgut epithelium leads to loss of infection in incompatible sand fly-*Leishmania* species combinations. In competent vectors, a substantial part of the parasite population survives defecation and colonizes the midgut. Finally the infection spreads anteriorly to be transmitted to the next host during subsequent blood meals [12].

Sand flies of the genus *Sergentomyia* are proven vectors of reptile *Leishmania* species, non-pathogenic to humans, which exhibit hypopylarian development patterns in their insect vectors (developing in the hindgut) with transmission occurring by predation of the infected flies by lizards. However, *Sergentomyia* species are not fully restricted to feeding on reptiles and at least some species feed on humans and/or mammalian reservoirs of *Leishmania* pathogenic to humans. Therefore, they have been suspected as vectors in some visceral leishmaniasis (VL) and cutaneous leishmaniasis (CL) foci where *Sergentomyia* spp. were abundant and found to harbour *Leishmania* (reviewed by [13]). Recently, a vectorial role was strongly suggested for three *Sergentomyia* species including *S*. *schwetzi* in the Mont-Rolland area in Senegal [14]. However, experiments showed that three species of *Leishmania* pathogenic to humans (*L*. *donovani*, *L*. *infantum* and *L*. *major*) did not survive defecation of bloodmeal remnants in *S*. *schwetzi* [15–17]. It was proposed that the crucial aspect mediating the refractoriness of *S*. *schwetzi* was the relative timing of degradation of the PM and defecation [17] i.e. the extremely long persistence of the PM in *S*. *schwetzi*, in comparison with three species of the genus *Phlebotomus* [18].

The aim of the current study was to compare the morphology and ultrastructure of the PM and activity of endogenous chitinase in *S*. *schwetzi* with three *Phlebotomus* species and to demonstrate that persistence of the PM represents an important mechanism of refractoriness of *S*. *schwetzi*. We showed that the PM of *S*. *schwetzi* shares similar morphological features with *Phlebotomus* species transmitting *Leishmania*. The role of the PM in refractoriness of *S*. *schwetzi* was demonstrated experimentally; the artificial disintegration of the PM using culture media from *B*. *bassiana*, rich in chitinase activity, enabled the development of mature infections of both *L*. *major* and *L*. *donovani* in *S*. *schwetzi* females.

## Methods

### Sand flies and *Leishmania*

Laboratory colonies of *Sergentomyia schwetzi* (from Ethiopia), *Phlebotomus orientalis* (from Ethiopia), *P*. *argentipes* (from India) and *P*. *papatasi* (from Turkey) were maintained in the insectary of the Charles University in Prague under standard conditions (at 26 °C fed on 50% sucrose with a 14 h light/10 h dark photoperiod) as described previously [19]. *Leishmania donovani* (MHOM/ET/2010/GR374) transfected with green fluorescent protein (GFP) were cultured in M199 medium (Sigma) containing 10% heat-inactivated fetal bovine serum (FBS, Gibco) supplemented with 1% BME vitamins (Basal Medium Eagle, Sigma), 2% sterile urine, 250 μg/mL amikacin (Amikin, Bristol-Myers Squibb) and 150 μg/mL selective antibiotic G418 (Sigma). *Leishmania major* LV561 (LRC-L137; MHOM/IL/1967/Jericho-II) transfected with GFP protein were cultured in the same medium without selective antibiotics.

### Origin of exogenous chitinases

Chitinases from *Streptomyces griseus* and *Trichoderma viride* (both from Sigma, Cat. No C6137 and C8241, respectively) were diluted in PBS buffer and stored at -20 ^o^C. *Beauveria bassiana* CCF 4422, obtained from the Culture Collection of Fungi (CCF, Dept. of Botany, Faculty of Science, Charles University, Prague, Czech Republic) was grown at 26 ^o^C in a medium composed of (w/v) glucose 5%, Neopeptone (BD) 5%, Yeast extract (Sigma) 1%, NaCl (0.25%) in distilled water (modified after [25]). After three weeks of cultivation, the whole volume was centrifuged at 250 g for 10 min, the supernatant was sterilized using 0.22 μm millipore filter units (Millex-GP), concentrated by centrifugation through 30 kDa filters (Amicon) and stored at -80 ^o^C.

### Sand fly infections

*Leishmania* promastigotes from log-phase cultures (day 3–4 post inoculation) were resuspended in heat-inactivated rabbit blood (MVDr. Zdenek Petr, Czech Republic) and mixed 1:1 with a supernatant from *B*. *bassiana* culture containing chitinase (preparation described above) to obtain final concentration 1x10^6^ promastigotes/mL and 0.07 U of exochitinase activity/mL. Control flies were fed on the same *Leishmania* suspension in blood mixed with the fresh culture medium or heat-inactivated culture supernatant from *B*. *bassiana*. Sand fly females (5–9 days old) were infected by feeding through a chick-skin membrane (BIOPHARM, Czech Republic) on the suspension. Engorged sand flies were maintained in the same conditions as the colony. Females were dissected in several time intervals post bloodmeal (PBM), the density and location of *Leishmania* infections in their digestive tract was examined by fluorescence microscopy. Parasite loads were graded according to [20] as light (< 100 parasites per gut), moderate (100–1000 parasites per gut) and heavy (> 1000 parasites per gut). Experiments with each *Leishmania*–sand fly combination were repeated four to six times.

### Morphometry of parasites

Midgut smears of sand flies infected with *Leishmania* parasites were fixed with methanol, stained with Giemsa, examined under the light microscope Olympus BX51 and photographed with an Olympus D70 camera. Body length, flagellar length and body width of parasites were measured using Image-J software. Four morphological forms were distinguished as described in [21]: procyclic promastigotes (PP), elongated nectomonads (EN), metacyclic promastigotes (MP) and short promastigotes (SP). Haptomonads were not distinguished as these forms remain attached on the gut and their numbers are often underestimated on gut smears. In total, 200 promastigotes from four females/smears were measured for each *Leishmania* species.

### Light microscopy of the PM

Bloodfed females were dissected at 10 intervals after feeding on anesthetized BALB/c mice, starting immediately post bloodmeal (PBM) and at each of the following times: 1, 3, 6, 12, 24, 48, 72, 96 and 120 h PBM. Dissections were carried out in the isotonic saline solution with brief washing of the gut in distilled water in order to better separate the PM [22]. For each sand fly species and time interval, at least 20 females were analysed. Slides were observed under an Olympus BX51 microscope with Nomarski contrast and photographed with an Olympus D70 camera and software. The colour of the PM and presence of the anterior plug (AP) and posterior ending of the PM were checked.

### Histology

Females at 24 h PBM were fixed at 4 ^o^C for 48 h in AFA solution (formaldehyde: ethanol: acetic acid: distilled water, 1.5:12.5:1:10). After washing in phosphate-buffered saline (pH 7.6) and dehydration in 70% to 96% ethanol, the samples were embedded in JB-4 resin following the manufacturer's instructions (Polysciences). Histological sections (2–6 μm thick) were stained with Ehrlich's acid haematoxylin and 0.2% eosin, mounted on glass slides with Euparal Mounting Medium (BioQuipProducts) and observed and photographed. Ten females of each sand fly species were used.

### Electron microscopy

Females were dissected at 24 h (*P*. *argentipes*) or 48 h PBM (*P*. *orientalis*, *P*. *papatasi*, *S*. *schwetzi*), fixed in modified Karnovsky´s fixative [23], and post-fixed with a 2% osmium tetroxide solution (both at 4 ^o^C). The samples were dehydrated in ascending concentration of ethanol (35–100%) then acetone (100%) and embedded in SPURR resin (SPI-chemo). Semithin sections (500 nm) and ultrathin sections (80–90 nm) were obtained using a Reichert-Jung Ultracut E ultramicrotome. Semithin sections were stained with toluidine blue for light microscopy. Ultrathin sections stained with uranyl acetate and lead citrate [24] were observed and photographed with a Jeol 1011 transmission electron microscope with iTEM 5.1 software (Olympus). Thickness of the PM was measured on representative images using the Image-J software. Together 400 measurements per each sand fly species were obtained (four females per species, 10 images per female and 10 measurements per image).

### Assessment of chitinase activity

The exochitinase activity of supernatants from *B*. *bassiana* culture (preparation described above) or dissected sand fly midguts was quantified using the Chitinase Assay Kit (Sigma, Cat. No. CS0980) by monitoring the hydrolysis of the chitinase substrates (4-Nitrophenyl N, N′-diacetyl-β-D-chitobioside and 4-Nitrophenyl N-acetyl-β-D-glucosaminide). Midguts were dissected at 24, 48 and 72 h PBM and pools of 20 midguts in 40 μL of TRIS-NaCl buffer (20 mM TRIS, 150 mM NaCl, pH 7.6) were used. Specimens were kept on ice during dissections and samples were processed immediately. Midgut pools were homogenized and centrifuged at 12 100 g for 10 min. The assay was performed in triplicates and according to instructions of the manufacturer. Briefly, 2 μL of supernatant were diluted in 98 μL of the substrate solution (1 mg of substrate dissolved in 1 mL of the Assay Buffer, Cat. No. A4855). Samples were incubated for 30 minutes at 37°C and pH 4.8. The absorption of the released 4-nitrophenol was measured after the addition of the Stop Solution (sodium carbonate solution) colorimetrically at 405 nm on INFINITE M200 spectrophotometer (Schoeller instruments). The exochitinase activity was calculated according to the manufacturer's instructions (one unit release 1 μmole of 4-nitrophenolfrom the appropriate substrate per minute at pH 4.8 and 37°C).

### Effect of chitinases from *S*. *griseus* and *T*. *viride* on *Leishmania* growth *in vitro*

*L*. *major* promastigotes (at concentration of 1x10^6^/mL) were exposed to chitinases from *S*. *griseus* and *T*. *viride* serially diluted in PBS in 96-well flat bottomed microtitre plates. Five μL of each dilution of chitinase were added to 200 μL of the *Leishmania* suspension in culture medium or defibrinated and inactivated rabbit blood. In controls, five μL of PBS were added. Numbers of parasites were counted after 24 h of cultivation at 26 ^o^C using the Burker cell counter.

### Effect of exogenous chitinases from *S*. *griseus* and culture supernatant from *B*. *bassiana* on *Leishmania* survival *in vitro*

*L*. *major* and *L*. *donovani* promastigotes at concentration of 1x10^6^/mL in a volume of 100 μL of culture medium were mixed with 100 μL of the supernatant from *B*. *bassiana* culture (prepared as described above) in 96-well flat-bottomed microtitre plates. The resultant concentration of chitinase was 0.07 U/mL (an identical concentration to that used for experimental infections of sand flies). As a control, 100 μL of the fresh culture medium for *B*. *bassiana* instead of the supernatant were added. For testing of the effect of *S*. *griseus* chitinase, 5 μL of chitinase solution in PBS was added to promastigotes at concentration of 1x10^6^/mL in a volume of 200 μL of culture medium to obtain chitinase concentration of 1 U/mL, 0.5 U/mL or 0.07 U/mL. As a control, 5 μL of PBS was added to the culture medium. Numbers of parasites were counted after 24 h of cultivation at 26 ^o^C using the flow cytometer CytoFLEX S (Beckman Coulter, Inc., Brea, California, USA) equipped with 4 lasers (405 nm, 488 nm, 561 nm, 638 nm) and 13 fluorescence detectors. Dead cells were marked with DAPI (4′, 6-Diamidine-2′-phenylindole dihydrochloride, 0.005 mg/mL; Thermo Fisher Scientific). The mortality was assessed as a ratio of the number of dead cells showing both green and blue fluorescence (GFP and DAPI) to the number of live cells showing green fluorescence (GFP). In samples where dead cells were completely disintegrated and total numbers of detected cells were significantly lower in comparison with control samples, mortality was counted using the total numbers of parasites in control wells. *Leishmania* promastigotes killed by solution of 1% formaldehyde in PBS and permeabilised with 0.5% Triton X-100 (Sigma) were used as a control for dead cells. GFP was excited using 488 nm laser and its fluorescence emission was detected using 525/40 filter, DAPI was excited by 405 nm laser and detected using 450/50 filter. Analysis of cytometry data was performed using CytExpert software (Beckman Coulter). The experiments were conducted in duplets and repeated 2–3 times.

### Detection of midgut O-glycosylated proteins

Midgut lysates from 3–5 days old females of *S*. *schwetzi* were separated by SDS PAGE (10% gel, reducing conditions) followed by blotting as described previously [26]. Midgut lysates of two *Phlebotomus* species with known glycosylation [26] served as negative (*P*. *papatasi*) and positive (*P*. *argentipes*) controls, respectively. The nitrocellulose membrane was blocked overnight at 4°C in 20 mM Tris, 150 mM NaCl, 0.05% Tween, pH 7.6 (TBS-Tw) with 5% bovine serum albumin. Then the membrane was incubated with biotinylated *Helix pomatia* lectin (HPA, 1.25 μg/mL) for 1 h. In the control groups, the HPA was preincubated with the carbohydrate inhibitor N-acetyl-galactosamine (GalNAc, 250 mM) for 30 min. After washing, blots were incubated with streptavidin peroxidase (2.5 μg/mL) for 1 h and developed in 4-chloro-1-naphtol solution.

### Statistical analysis

Measurements of parasites and mortality of parasites *in vitro* were compared using Analysis of Variance (ANOVA) including Tukey Post Hoc Test, measurements of the thickness of the PM were tested with Nested ANOVA and Post Hoc Test (t–test with Bonferroni-Holm correction). Differences in defecation between chitinase treated flies and control group were analysed by proportional test with Holm-Bonferroni correction. All the statistical evaluations were performed with statistical software SPSS version 23 or R software (http://cran.r-project.org).

## Results

### Morphology and ultrastructure of the PM in four sand fly species

**Fig 1:**
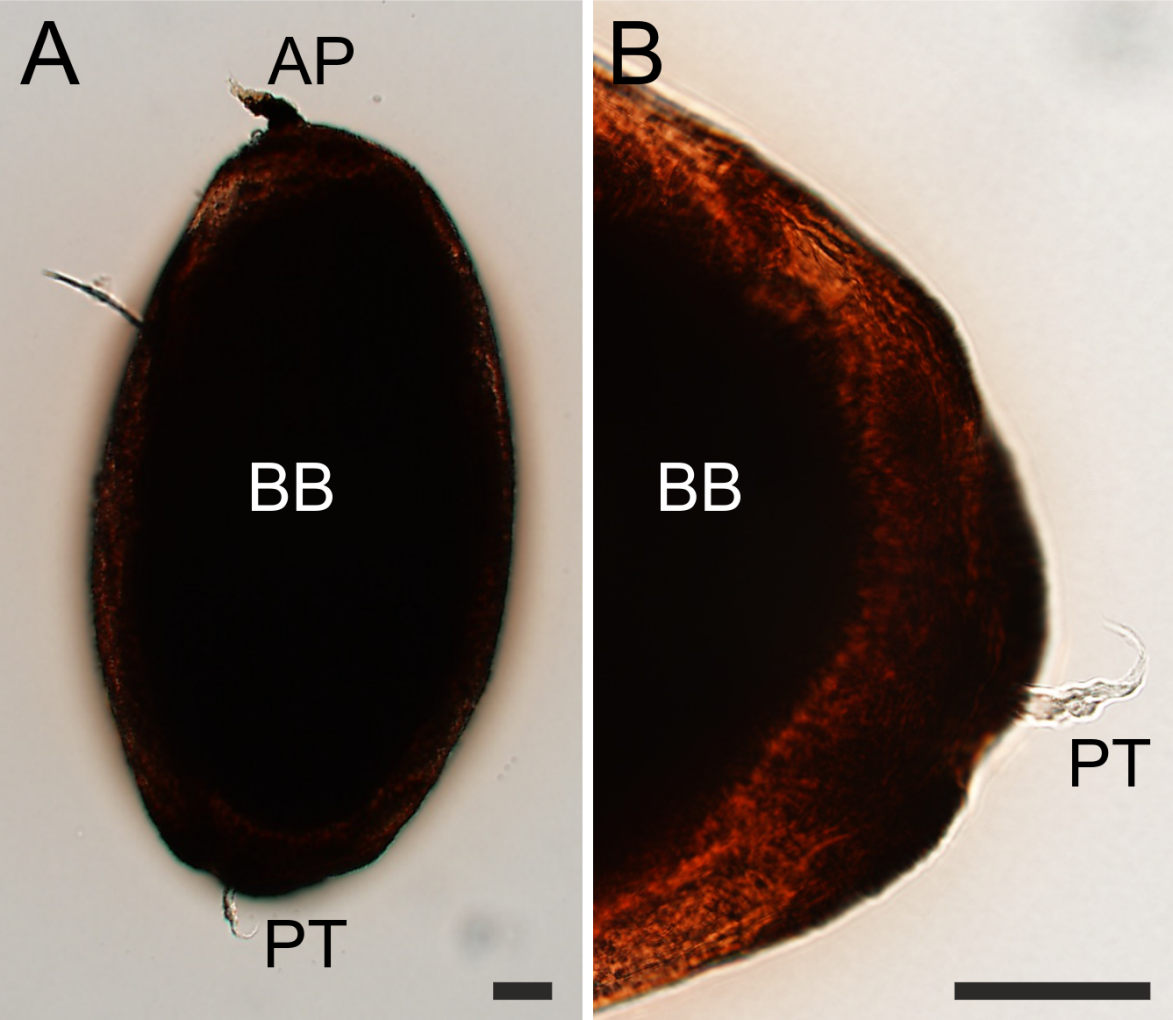
Gross morphology of the *S*. *schwetzi* PM with detailed image of the posterior tail. The complete PM dissected from the gut of *S*. *schwetzi* at 24 h PBM (A) and its enlarged posterior end with the transparent posterior tail showed in more detail (B). PT, posterior tail; AP, anterior plug; BB, blood bolus. Scale bars indicate 100 μm.

Morphology and ultrastructure of the PM of *S*. *schwetzi* was compared with three species of the genus *Phlebotomus*: *P*. *orientalis*, *P*. *argentipes* and *P*. *papatasi* to ascertain if there are specific morphological traits which may be connected with the long persistence of the PM observed in *S*. *schwetzi* [18]. Light microscopy and analysis of sections from specimens embedded in JB-4 resin showed that these four sand fly species did not differ substantially in the gross morphology of the PM. The anterior plug, i.e. the most anterior part of the PM secreted by the thoracic midgut, was formed in all the four sand fly species (S1 Fig). The PM was evenly closed on the posterior end, usually forming a short funnel-shaped closed structure called the posterior tail (Fig 1). In all the four species, the PM was originally transparent and then darkened as it became encrusted by heme, this process was apparent beginning 24 h PBM. The only parts of the PM which remained permanently transparent were the AP and the posterior tail.

**Fig 2:**
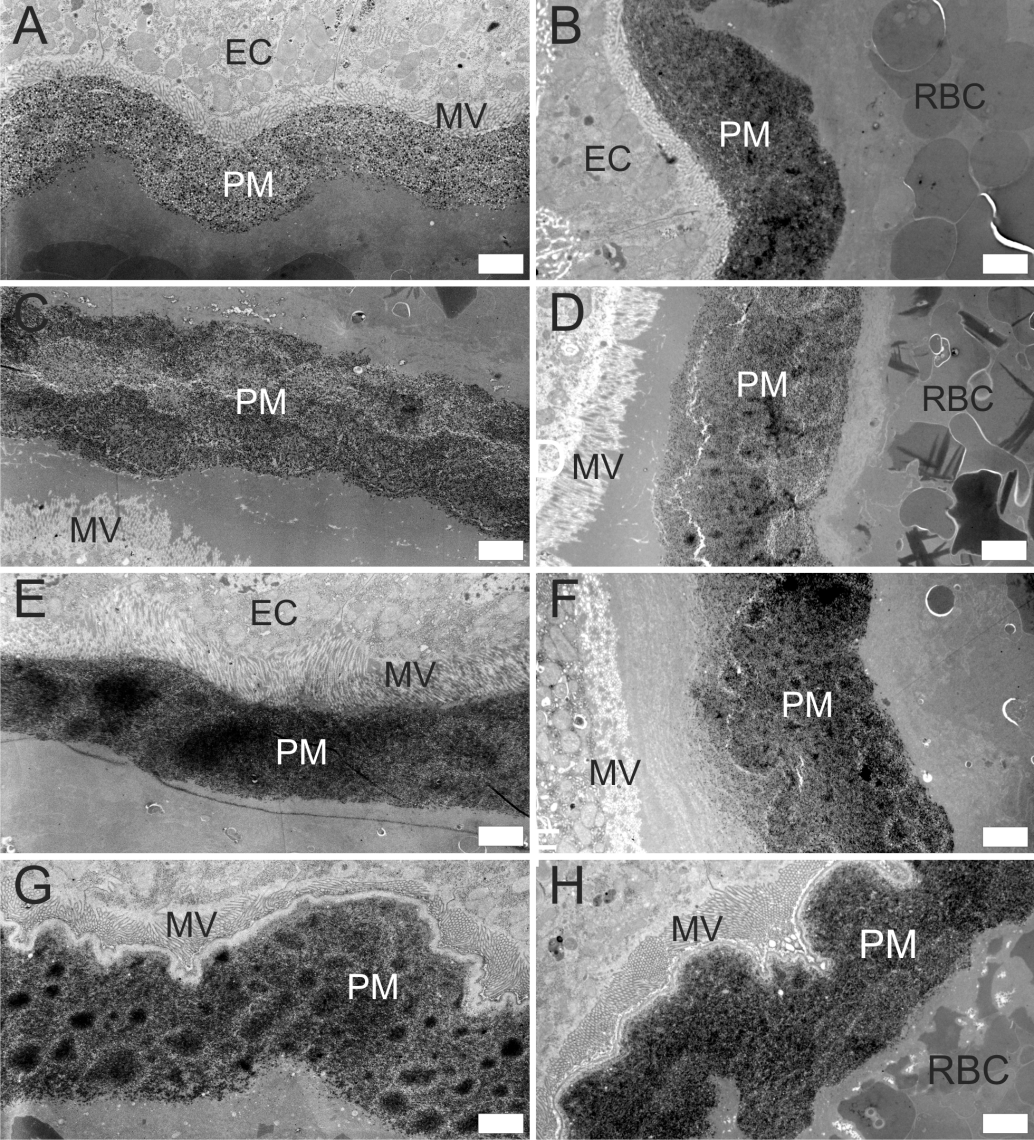
Ultrastructure of the PM in four sand fly species. Electron micrographs of cross sections of the AMG in *P*. *argentipes* (A, B); *P*. *orientalis* (C, D); *P*. *papatasi* (E, F) and *S*. *schwetzi* (G, H) dissected at 24 h (*P*. *argentipes*) or 48 h PBM (*P*. *orientalis*, *P*. *papatasi*, *S*. *schwetzi*). PM, peritrophic matrix; MV, microvilli; EC, epithelial cells of the AMG. Scale bars indicate 2 μm.

Thickness and ultrastructure of the mature PM was assessed using images from electron microscopy. Based on knowledge of the PM kinetics [18], the optimal time for evaluation of the mature PM was 48 h PBM in *P*. *orientalis*, *P*. *papatasi* and *S*. *schwetzi* and 24 h PBM in *P*. *argentipes* (degradation of the PM proceeds faster in *P*. *argentipes*, before 48 h PBM [18]). The mature PM of *S*. *schwetzi* was as thick as those of *P*. *orientalis* and *P*. *papatasi*, while the PM of *P*. *argentipes* was significantly thinner than the PM of these three sand fly species (Table 1). This result corresponds with observations made during dissections of fed females: the PM of *P*. *argentipes* was extremely fragile and its extraction from the midgut was difficult. The ultrastructure of the mature PM also differed between *P*. *argentipes* and the remaining three sand fly species. While the mature PM consisted of thin solid outer layer and thick granular inner layer in *S*. *schwetzi*, *P*. *orientalis* and *P*. *papatasi*, the PM of *P*. *argentipes* appeared fairly homogeneous throughout the entire cross-section (Fig 2). Thus, the PM of *S*. *schwetzi* did not show any unique morphological features in comparison with the other two vectors of the genus *Phlebotomus*.

**Table 1:** Thickness of the PM in four sand fly species measured from TEM images.

Sand fly species	Measurements of the PM thickness				Significance of between-species differences^a^			
	No.	Median	Mean (S.D.) (μm)	Range (μm)	*P*. *argentipes*	*P*. *orientalis*	*S*. *schwetzi*	*P*. *papatasi*
*P*. *argentipes*	400	3.74	3.87 (1.11)	1.54–7.94	-			
*P*. *orientalis*	400	7.39	7.22 (1.73)	3.41–13.76	P = 0.007	-		
*S*. *schwetzi*	400	6.49	6.68 (1.97)	2.65–12.87	P = 0.016	P = 1.000	-	
*P*. *papatasi*	400	6.78	7.16 (2.11)	2.58–15.19	P = 0.007	P = 1.000	P = 1.000	-

^a^Significance of between-species differences was tested by Nested ANOVA and post Hoc Test (t–test with Bonferroni-Holm correction).

### Midgut chitinase activity

**Fig 3:**
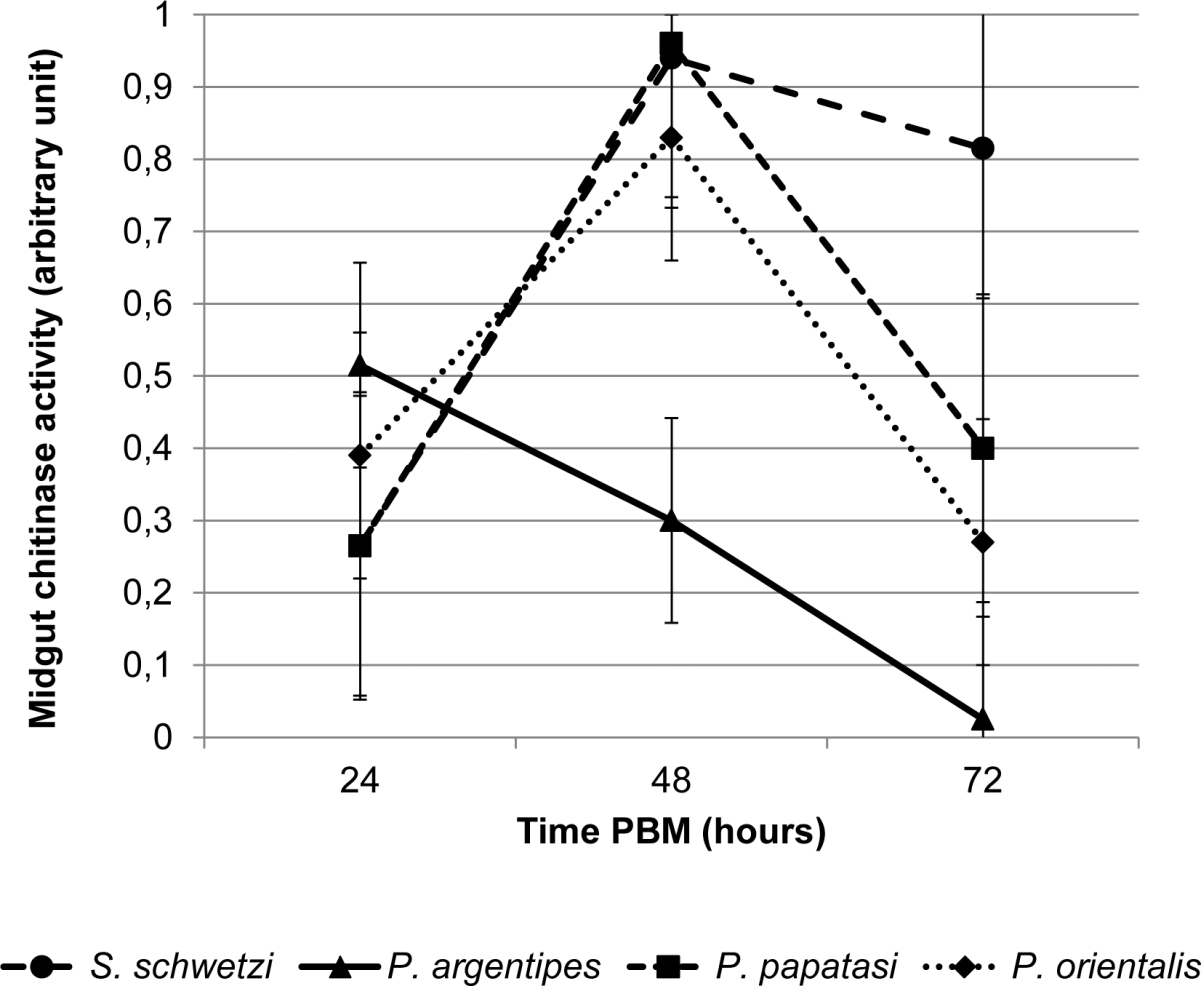
Chitinase activity in midguts of the four sand fly species. The exochitinase activity was quantified using the Chitinase Assay Kit (Sigma) in dissected sand fly midguts at 24, 48 and 72 h PBM. One arbitrary unit of chitinase releases 1 μmole of 4-nitrophenol per minute from the appropriate substrate at pH 4.8 at 37 °C. The values are arithmetic means from two experiments.

Next we tested chitinase activity in midguts of the four sand fly species to determine whether the long persistence of the PM in *S*. *schwetzi* might be caused by lower activity of midgut chitinase in this species. Interestingly, the dynamics and levels of exochitinase activity of *S*. *schwetzi* were similar to those in *P*. *papatasi* and *P*. *orientalis*: activity was detectable at 24 h PBM, peaked at 48 h PBM and then decreased at 72 h PBM (Fig 3). Moreover, the activity levels observed in *S*. *schwetzi* by 72 h PBM were the highest among all four sand fly species studied. A distinct course of exochitinase activity was observed in *P*. *argentipes*: the activity peaked at 24 h PBM, decreased by 48 h and declined almost to zero by 72 h PBM. Hence, the long persistence of the PM in *S*. *schwetzi* cannot be explained by low exochitinase activity in the midgut.

### Effect of exogenous chitinases on the survival of *Leishmania* promastigotes *in vitro*

**Fig 4:**
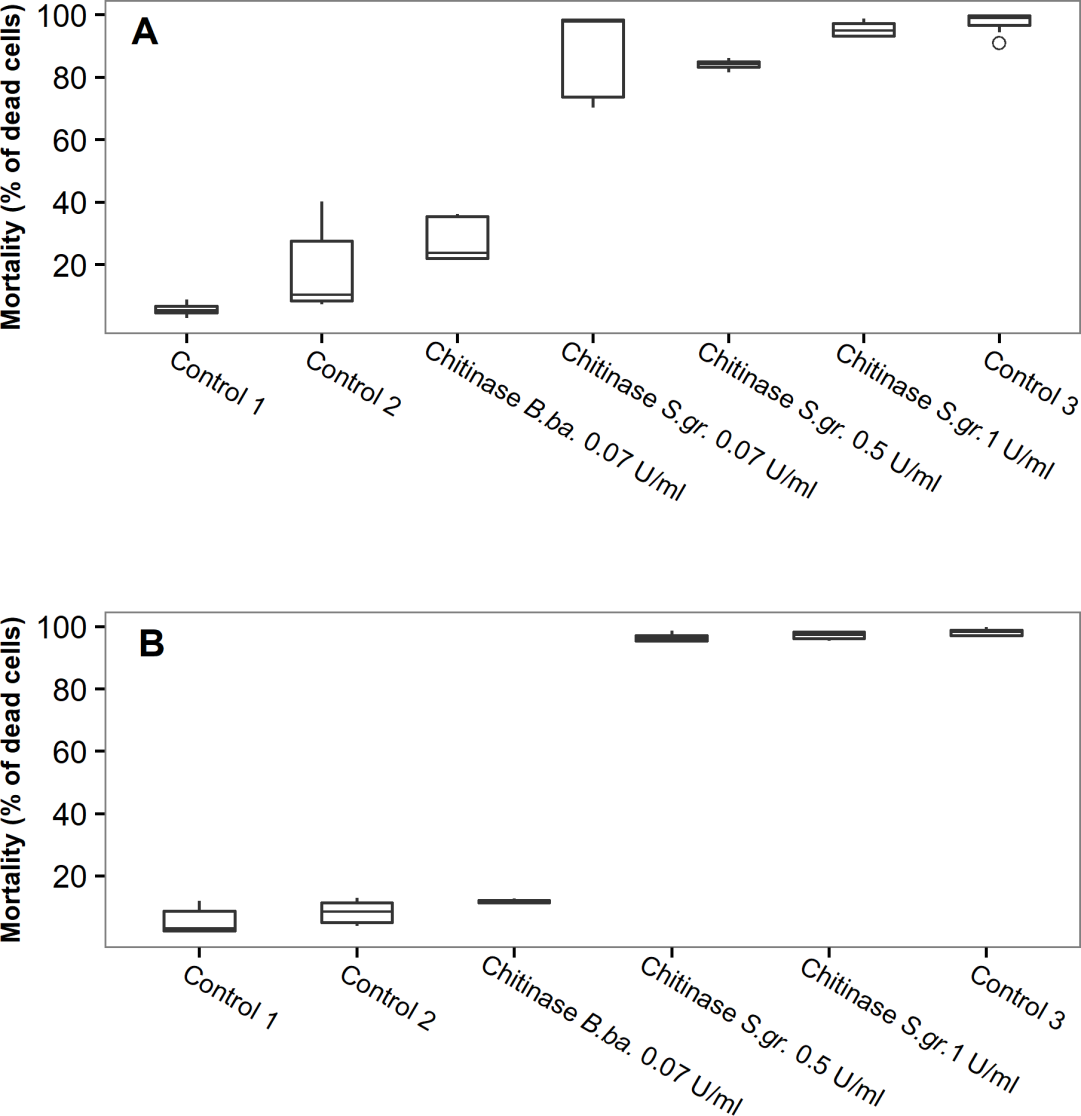
**Mortality of *L*. *donovani* (A) and *L*. *major* (B) promastigotes exposed *in vitro* to culture supernatant of *B*. *bassiana* or chitinase from *S*. *griseus*.** The mortality assessed as the ratio of the number of dead cells showing both green and blue fluorescence (GFP and DAPI) to the number of live cells showing green fluorescence (GFP). Control 1, culture medium for *Leishmania* with 0.025 V of PBS; Control 2, culture medium for *Leishmania* mixed at 1:1 with culture medium for *B*. *bassiana*; Control 3, parasites killed by 1% solution of formaldehyde and permeabilised by 0.5% Triton X-100; *B*. *ba*., *Beauveria bassiana*; *S*. *gr*., *Streptomyces griseus*.

Before feeding exogenous chitinase to *Leishmania*–infected sand flies, we had to prove that chitinase has no lethal effect on the parasites. However, preliminary experiments showed that both commercially available chitinases from *S*. *griseus* and *T*. *viride* significantly decreased growth or even killed *Leishmania* promastigotes *in vitro* at all concentrations tested. (S1 Table). Therefore, we searched for a nontoxic source of chitinase and used the supernatant from the culture of the fungus *B*. *bassiana*. Flow cytometry was used to study the effect on *Leishmania* parasites *in vitro*. Supernatant from the culture of *B*. *bassiana* containing chitinase at concentration of 0.07 U/mL had no lethal effect on *L*. *major* and *L*. *donovani* promastigotes-mortality was very low and statistically not different from values observed for controls cultures without chitinase (Fig 4, P = 0.505 and P = 0.559 for *L*. *major* or *L*. *donovani*, respectively, ANOVA and Tukey Post Hoc Test). On the other hand, flow cytometry confirmed the lethal effect of chitinase from *S*. *griseus* on promastigote growth in cultures (S1 Table)-it killed *L*. *major* and *L*. *donovani* at all tested concentrations in the same rate as did the negative control (parasites killed by 1% solution of formaldehyde) (Fig 4, P > 0.05 for all chitinase concentrations and both *Leishmania* species, ANOVA and Tukey Post Hoc Test).

### Effect of culture supernatant from *B*. *bassiana* on the PM of *S*. *schwetzi* and development of *Leishmania* parasites *in vivo*

**Fig 5:**
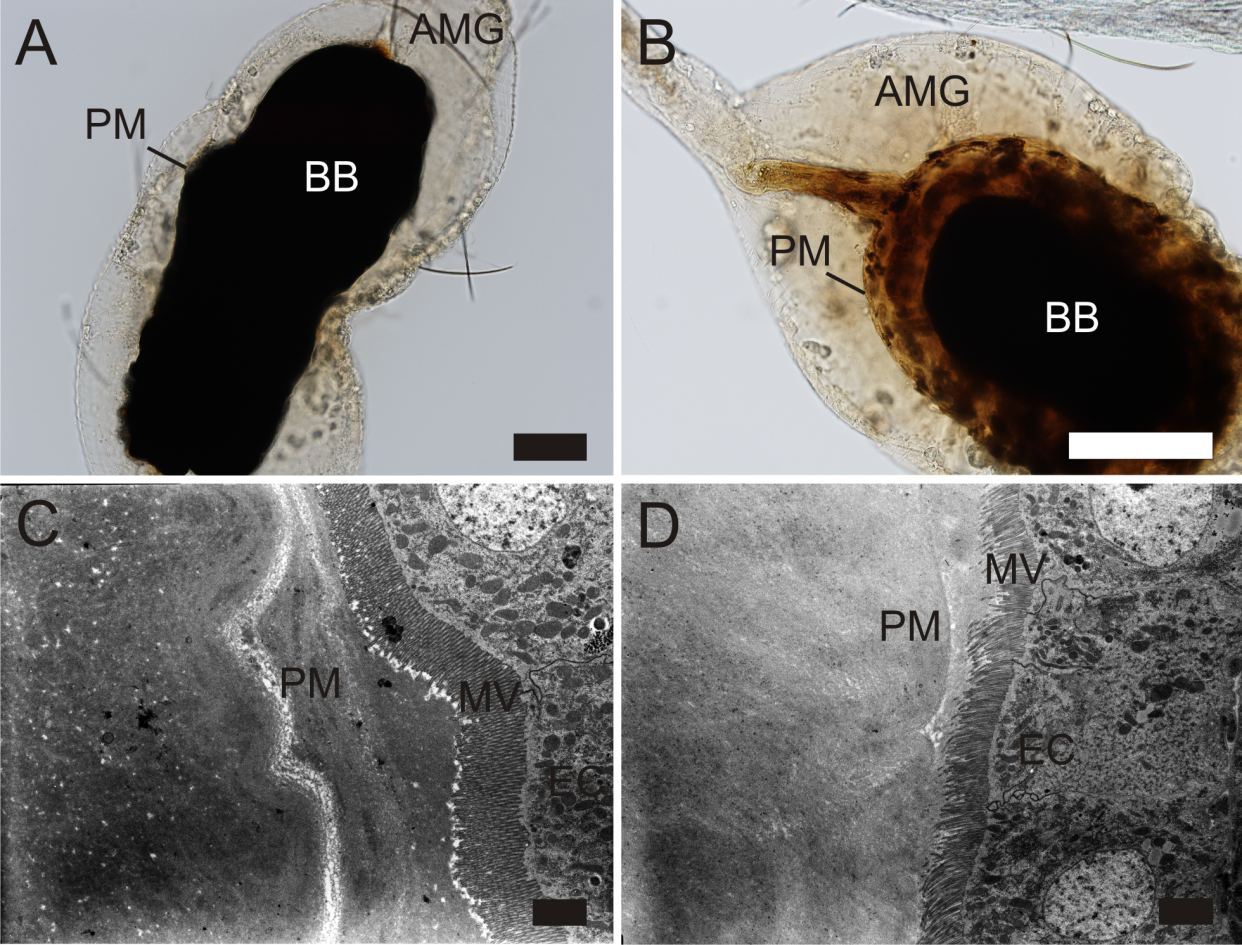
Differences in the PM between *S*. *schwetzi* females treated with *B*. *bassiana* culture supernatant and control. A and B, light microscopy; C and D, electron micrographs of cross sections of the abdominal midgut. A, appearance of the PM in a control female on day 3 PBM; B, appearance of the PM in female fed on a mixture of rabbit blood with supernatant of *B*. *bassiana* containing chitinase on day 3 PBM; C, multilayered thick PM in the control female on day 2 PBM; D, thin PM without apparent inner structure in the female fed on a mixture of rabbit blood with supernatant of *B*. *bassiana* containing chitinase on day 2 PBM. AMG, abdominal midgut; BB, blood bolus; PM, peritrophic matrix; MV, microvilli; EC, epithelial cells of the AMG. Scale bars indicate 100 μm in A, B and 2 μm in C, D.

Females of *S*. *schwetzi* were membrane-fed on a mixture of blood with culture supernatant from *B*. *bassiana* containing chitinase to disrupt the PM and mimic early disintegration of the PM occurring in most *Phlebotomus* species. Light microscopy 24 h PBM revealed that the PM was disintegrated in 94% of chitinase-treated females (N = 49) while no disintegration was observed in control females (N = 35). By day 3 PBM the PM in control flies was consistently dark and opaque due to incrustation with heme (Fig 5A) while the PM in chitinase-treated flies remained mostly transparent with patches of dark coloration (Fig 5B). Presence of red blood cells in the ectoperitrophic space was indicative of partial disintegration and increased permeability of the PM in chitinase-treated females.

Electron micrographs of cross sections of the AMG on day 2 PBM confirmed the differences at the structural level. While the PM in control flies was thick and multilayered (Fig 5C), addition of *B*. *bassiana* culture supernatant to the bloodmeal resulted in thin PMs without apparent inner structure, only scarcely distinguishable from the background (Fig 5D). Surprisingly, although the PM in flies treated with the culture supernatant was weakened and permeable, it maintained its structure in midguts longer than controls (even till day10 PBM) as the defecation in this group was significantly delayed (S2 Fig).

**Fig 6:**
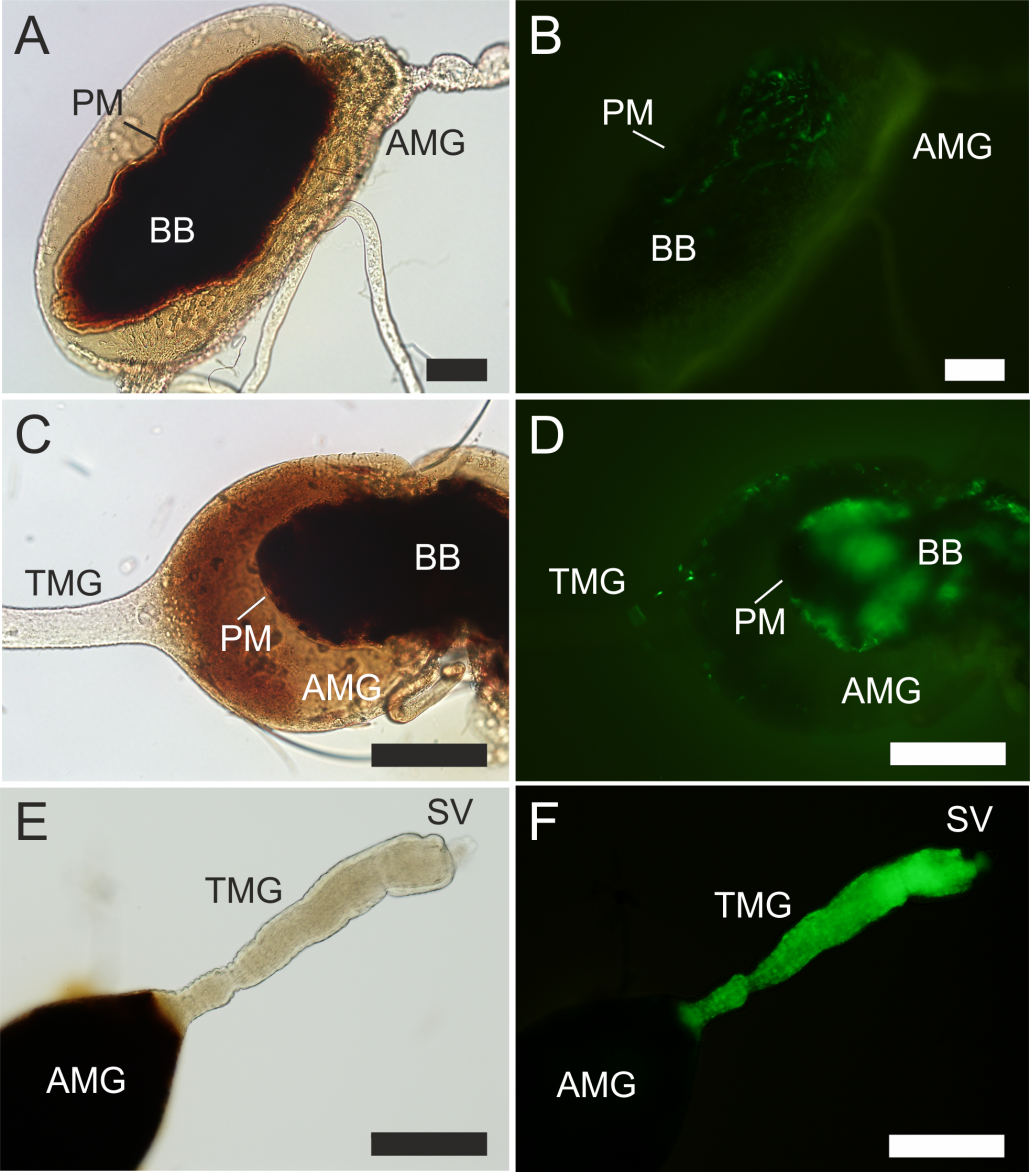
Light and fluorescent microscopy of *S*. *schwetzi* midguts. A and B, the gut of a control female (infectious meal comprising *L*. *donovani* promastigotes suspended in a mixture of fresh *B*. *bassiana* medium and inactivated rabbit blood) on day 3 PBM with *L*. *donovani* enclosed inside the PM; C and D, the gut of a treated female (infectious meal comprising *L*. *donovani* promastigotes suspended in a mixture of *B*. *bassiana* culture supernatant and inactivated rabbit blood) on day 3 PBM showing escape of *L*. *donovani* from the endoperitrophic space; E and F, the gut of a treated female on day 10 PBM showing colonization of the thoracic midgut and the stomodeal valve by *L*. *major* promastigotes expressing GFP (green). Images A—B, C—D and E—F are the same guts photographed by light and fluorescent microscopy, respectively. Both *Leishmania* species were marked with GFP protein, the midgut epithelium shows a natural mild autofluorescence. AMG, abdominal midgut; TMG, thoracic midgut; BB, blood bolus; SV, stomodeal valve; PM, peritrophic matrix. Scale bars indicate 100 μm.

**Fig 7:**
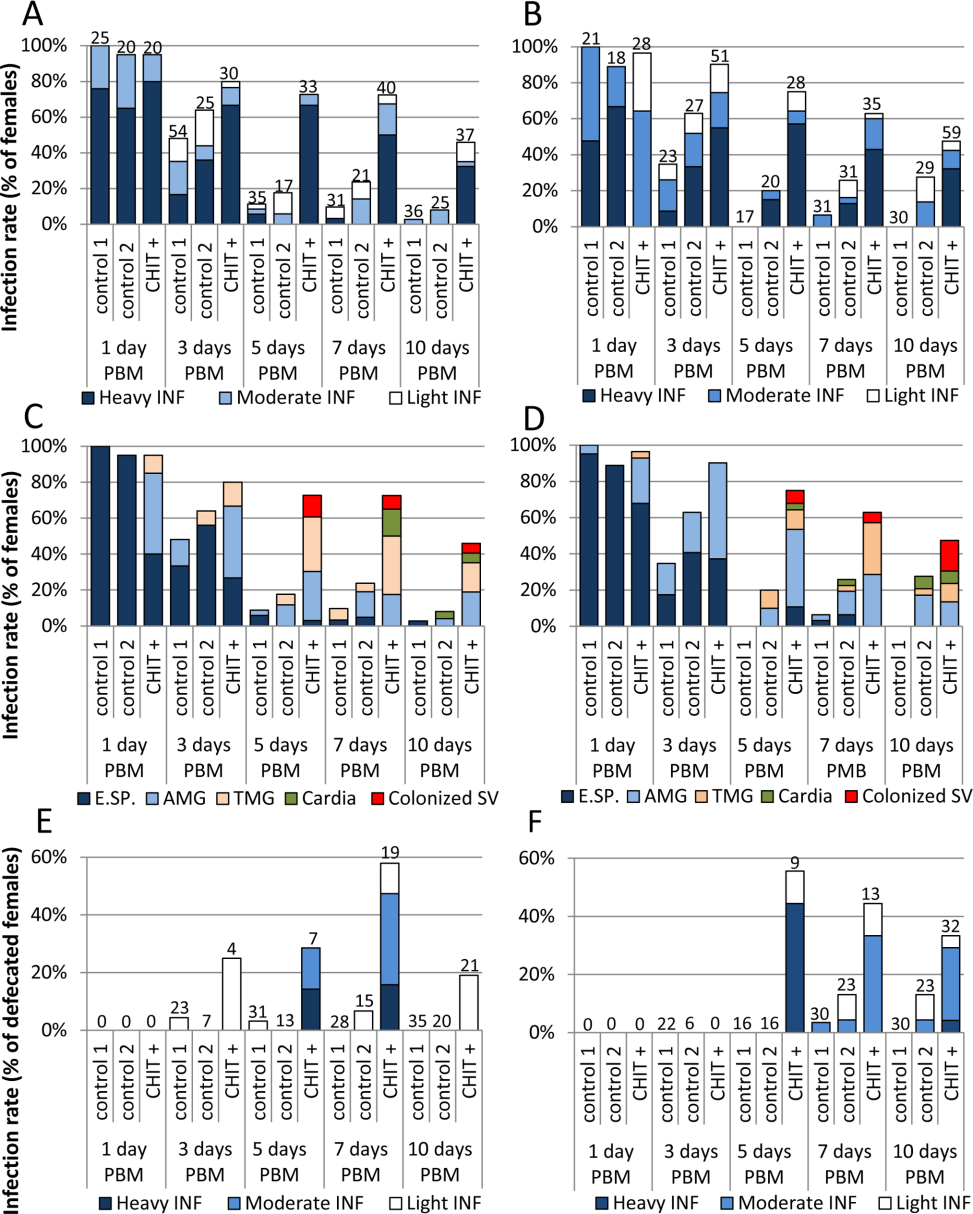
Effect of *B*. *bassiana* culture supernatant on the development of *L*. *donovani* and *L*. *major* in *S*. *schwetzi*. Experimental infections of *S*. *schwetzi* with *L*. *donovani* (A, C, E) and *L*. *major* (B, D, F) with addition of culture supernatant containing exogenous chitinase (0.07 U/mL) from *B*. *bassiana* (CHIT +). In control females, either fresh medium for *B*. *bassiana* or heat-inactivated supernatant from *B*. *bassiana* culture was used (control 1 and control 2, respectively). A, B: Rates and intensities of infections. Numbers of dissected females are shown above bars. C, D: Location of *L*. *donovani* in infected sand flies. E.SP., endoperitrophic space; AMG, abdominal midgut; TMG, thoracic midgut; SV, stomodeal valve. E, F: Rates and intensities of infections in females post defecation. Numbers of dissected females post defecation are shown above bars.

The same culture supernatant from *B*. *bassiana* with 0.07 U/mL of chitinase was used for experimental infections of *S*. *schwetzi* with *Leishmania* parasites. Addition of culture supernatants significantly enhanced development of *L*. *donovani* and *L*. *major* in *S*. *schwetzi*. In control females fed on heat-inactivated culture supernatants or fresh culture media mixed with rabbit blood, neither species of *Leishmania* were able to develop mature infections, only very exceptionally survived defecation and then the infections were lost. On the other hand, in groups treated with *B*. *bassiana* culture supernatants, both *Leishmania* species escaped from the PM (Fig 6A and 6B), developed heavy infections in most females (Fig 7A and 7B) and by day 10 PBM colonized the stomodeal valve in 12% and 36% of females infected with *L*. *donovani* and *L*. *major*, respectively (Fig 6C and 6D and Fig 7C and 7D). Moreover, analysis of morphological forms revealed the presence of metacyclic promastigotes in late infections of both *Leishmania* species (Table 2).

**Table 2:** The relative representation and measurements of three morphological forms of *L*. *major* and *L*. *donovani* in guts of *S*. *schwetzi* by days 7–10 PBM.

*Leishmania* species	Promastigote form	N (%)	Body length (μm) Mean (S.D.) [Range]	Body width (μm) Mean (S.D.) [Range]	Flagellar length (μm) Mean (S.D.) [Range]
*L*. *major*	Elongated nectomonads	161 (80.5)	19.2 (2.9) [14.4–27.2]	1.4 (0.4) [0.7–3.4]	19.2 (3,9) [6.7–33.9]
	Short promastigotes	32 (16.0)	11.7 (1.8) [6.3–13.6]	1.9 (0.7) [1.0–3.7]	14.3 (4.2) [2.7–23.2]
	Metacyclic promastigotes	7 (3.5)	8.0 (1.6) [5.9–10.0]	2.3 (0.7) [1.1–3.0]	17.1 (2.5) [13.0–20.0]
	Total	200 (100)	17.6 (4.3) [5.9–27.2]	1.5 (0.6) [0.7–3.7]	18.3 (4.3) [2.7–33.9]
*L*. *donovani*	Elongated nectomonads	117 (58.5)	17.5 (2.4) [14.0–24.6]	1.6 (0.5) [0.7–4.7]	17.8 (3,1) [11.3–26.5]
	Short promastigotes	73 (36.5)	11.2 (1.8) [5.5–13.8]	1.6 (0.5) [0.9–3.3]	14.9 (3.9) [2.9–22.4]
	Metacyclic promastigotes	10 (5.0)	8.4 (2.0) [5.2–10.6]	1.6 (0.3) [1.1–2.1]	18.4 (3.4) [12.9–22.7]
	Total	200 (100)	14.8 (4.0) [5.2–24.6]	1.6 (0.5) [0.7–4.7]	16.8 (3.7) [2.9–26.5]

Sand flies were infected with addition of culture supernatant from *B*. *bassiana* (chitinase activity 0.07 U/mL).

Despite colonization of the ectoperitrophic space, the infection rates in females treated with culture supernatants gradually decreased from more than 90% on day 1 PMB to 46% and 47% in *L*. *donovani* and *L*. *major*, respectively, on day 10 PBM. In sand flies post defecation, infection rates ranged usually between 20% and 30% in both *Leishmania* species (Fig 7E and 7F) and attachment of parasites to the midgut epithelium was never observed.

### Glycosylation of the midgut epithelium

Midgut mucins bearing O-linked glycosylation with terminal N-acetyl-galactosamine have been previously demonstrated to participate in *Leishmania* attachment in so called permissive sand fly vectors [6,26,27]. Therefore, midgut lysate of *S*. *schwetzi* was subjected to SDS-PAGE followed by blotting with HPA lectin specific for N-acetyl-galactosamine. Midgut lysates of *P*. *argentipes* and *P*. *papatasi* served as a positive and negative control, respectively [26]. In *S*. *schwetzi* midgut lysate, lectin HPA showed specific reactivity with a molecule of apparent molecular mass around 35–40 kDa; however, the reaction was weak in comparison with *P*. *argentipes*. Control strips with inhibitory sugar added were negative, confirming the specificity of HPA binding (S3 Fig).

## Discussion

We demonstrated that the structure and thickness of the PM of *S*. *schwetzi* is very similar to those of *P*. *orientalis* and *P*. *papatasi*. It consists of a thin solid outer layer and an amorphous thick inner layer which correspond with data on the ultrastructure of PMs of several other species of the genera *Phlebotomus* and *Lutzomyia* [11,28–30]. On the other hand, the PM of *P*. *argentipes* was thin and homogeneous without distinct outer and inner layers which may be related to the exceptionally fast blood digestion described for this sand fly species [18]. The gross morphology of the PM of *S*. *schwetzi* also resembled other tested sand fly species: all four species had an anterior plug secreted by the thoracic midgut repeatedly detected in sand flies [11,29–31]. The posterior end of the PM was closed, with posterior tail similar to that observed previously in *P*. *duboscqi* and *P*. *papatasi* [11,32].

As we did not find significant differences between the PM of *S*. *schwetzi* and PMs of other sand fly species we tested the hypothesis that the persistence of the PM of *S*. *schwetzi* could be caused by lower activity of midgut chitinases. Surprisingly, experimental data suggest the opposite; the exochitinase activity in *S*. *schwetzi* midguts was the highest of the four sand fly species tested. Chitin degradation in insects is catalysed by various chitinolytic enzymes, for example in mosquitoes, chitinolysis is controlled by at least two distinct chitinases (endo- and exochitinase) and β-N-acetylglucosaminidase [33]. Therefore, we cannot exclude the possibility that low activity of enzymes other than exochitinase might be responsible for delayed degradation of PM in *S*. *schwetzi*. High exochitinase activity in *S*. *schwetzi* midguts may be connected with the fact that chitinolytic activities of chitinases are related not only to degradation, but are involved also in synthesis and modulation of the PM [33].

Experiments described here and our previous studies [17,18] were performed with *S*. *schwetzi* from the colony originating in north-western Ethiopia and infected with *L*. *donovani* parasites isolated in the same region. Previously, *S*. *schwetzi* originating from Kenya were showed to be refractory to local *L*. *major* and *L*. *donovani* [15,16]. The origin of sand flies and parasites is important mainly in context of recent findings from the Mont-Rolland region in Senegal, where *L*. *infantum* DNA was found in 4.19% of captured *S*. *schwetzi* females and living parasites were observed in anterior midgut of a single female without a bloodmeal [34]. This fact evokes the question as to what extent is the vector competence of *S*. *schwetzi* population—specific. This species shows a substantial morphological variability and two morphological forms of *S*. *schwetzi* ("typical" and "atypical") were found in Senegal [35,36], a large area from Sudan to West Africa [37], Uganda [37] and Kenya [38]. Therefore, revision of the taxonomy of *S*. *schwetzi* and further experimental studies with sand flies and *Leishmania* parasites originating from different areas would be very interesting. In addition, it is not clear if the prolonged persistence of the PM is the common feature of the genus *Sergentomyia*. Previously the PM was studied in a single member of this genus; *S*. *arpaklensis* (corresponds to *S*. *sintoni* based on recent nomenclature). Its PM did not break down with the end of digestion and was defecated intact [39–41], presumably causing refractoriness for the reptile pathogen *L*. *gymnodactyli* [40].

Importance of the PM in vector competence was supported experimentally by several authors. Addition of a chitinase inhibitor allosamidin to the infective bloodmeal led to entrapment of *L*. *major* within the PM of *P*. *papatasi* [10]. Similarly, silencing of the gene for PpChit 1, a sand fly-derived chitinase involved in degradation of the PM [42], reduced *L*. *major* load in midguts of *P*. *papatasi* [43] while knockdown of the PpPer1 gene (peritrophin involved in the formation and scaffolding of the PM [42]) led to increase in the parasite load [44].

Here, the addition of the supernatant from *B*. *bassiana* culture with chitinase activity to the bloodmeals of *S*. *schwetzi* led to disintegration of the PM which enabled survival of *Leishmania* parasites in *S*. *schwetzi*. Both *L*. *donovani* and *L*. *major* developed heavy infections in flies treated with culture supernatants containing chitinase, with most important prerequisites for parasite transmission to the next host: presence of metacyclic forms and colonization of the stomodeal valve. The relatively low concentration of chitinase (0.07 U/mL in comparison with 1 U/mL in the experiment of Pimenta et al. (1997)) [10] caused only partial disintegration of the PM. The colour of the PM was more transparent than in control flies which might indicate lower incrustation of the PM with heme. In experiments of Pimenta et al. (1997) [10], the absence of PM caused by addition of exogenous commercial chitinase to the bloodmeal of *P*. *papatasi* was associated with the total loss or 20% reduction of amastigote- and promastigote-initiated *L*. *major* infections, respectively. Authors suggested that the lack of the PM exacerbated lethal conditions as a result of proteinase activity in the blood-fed midgut.

Partial degradation of PM due to culture supernatants of *B*. *bassiana*, together with delayed defecation, enabled *Leishmania* parasites to establish the infection in the midgut of *S*. *schwetzi*. However, we did not observe the attachment of promastigotes to the midgut epithelium and infection rates decreased with time post bloodmeal. This could be explained by the shortage of ligand molecules in the midgut as O-glycosylated epitopes in *S*. *schwetzi* midgut were present in very low amounts, significantly lower than in permissive species *P*. *argentipes* [26]. The presence of O-glycosylated mucins in a permissive vector *P*. *argentipes* as well as their absence in *P*. *papatasi*, a specific vector of *L*. *major*, is in accordance with previously described data [26]. Other factors, like antimicrobial peptides [45, 46] may also negatively influence the establishment of *Leishmania* infections in the gut of *S*. *schwetzi*.

The results of the present study clearly confirm that the prolonged persistence of the PM till defecation contributes significantly to the refractoriness of Ethiopian *S*. *schwetzi* to *Leishmania* parasites.

## Supporting information

S1 Fig Gross morphology of the PM in four sand fly species. Guts of *S*. *schwetzi* (A, B), *P*. *argentipes* (C, D), *P*. *papatasi* (E, F) and *P*. *orientalis* (G, H) were dissected and photographed using the light microscope with DIC at 24 h PBM (A, C, E, G). Sections of sand flies embedded in JB-4 resin were stained with haematoxylin and eosin (B, D, F, and H). Large arrows indicate the anterior plug; small arrows indicate the PM. Scale bars indicate 100 μm.
(TIF)

S2 Fig Effect of chitinase addition on defecation of *S*. *schwetzi* females. Defecation of sand flies fed on mixture of inactivated rabbit blood mixed 1:1 with supernatant from the culture of *B*. *bassiana* containing chitinase (black squares) was compared with defecation on control females fed on the mixture of inactivated rabbit blood mixed 1:1 with medium for *B*. *bassiana* instead of the supernatant (open circles). Defecation status was assessed under the light microscope. Numbers of females were: 48, 81, 61, 75 and 96 for chitinase-treated group and 46, 77, 51, 62 and 66 for the control group in days 1, 3, 5, 7 and 10 PBM, respectively. The between–groups differences were significant by days 3–10 PBM (P < 0.05, tested by proportional test with Holm-Bonferroni correction).
(DOCX)

S3 Fig Detection of the GalNAc-containing glycoconjugates in three sand fly species. Gut homogenates were fractionated on SDS-PAGE, transferred to nitrocellulose membrane and incubated with biotinylated *Helix pomatia* lectin (HPA) specifically binding GalNAc. PAP, *P*. *papatasi*; ARG, *P*. *argentipes*; SER, *S*. *schwetzi*; +, separated gut homogenate incubated with lectin HPA; -, incubation of the homogenate with HPA preincubated with specific GalNAc.
(TIF)

S1 Table Effect of chitinases from *S*. *griseus* and *T*. *viride* on *Leishmania* growth *in vitro*. (DOCX)
