# Supplementary figures and images for: Refractoriness of Sergentomyia schwetzi to Leishmania spp. is mediated by the peritrophic matrix
Source: PLoS Negl Trop Dis. 2018 Apr 4;12(4):e0006382. doi: 10.1371/journal.pntd.0006382 (PMC5902042; doi:10.1371/journal.pntd.0006382)

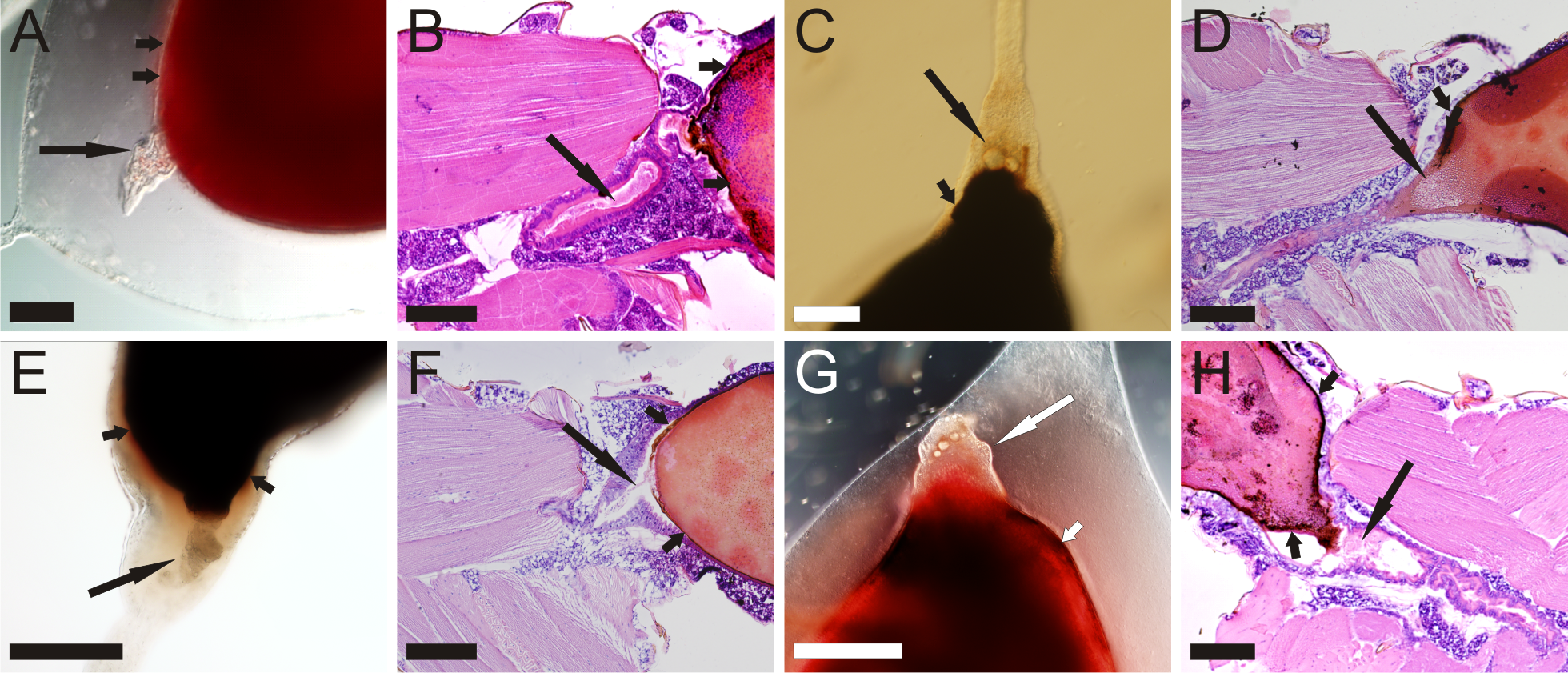

Supplement: S1 Fig — Guts of S. schwetzi (A, B), P. argentipes (C, D), P. papatasi (E, F) and P. orientalis (G, H) were dissected and photographed using the light microscope with DIC at 24 h PBM (A, C, E, G). Sections of sand flies embedded in JB-4 resin were stained with haematoxylin and eosin (B, D, F, and H). Large arrows indicate the anterior plug; small arrows indicate the PM. Scale bars indicate 100 μm. (TIF) [file pntd.0006382.s001.TIF]

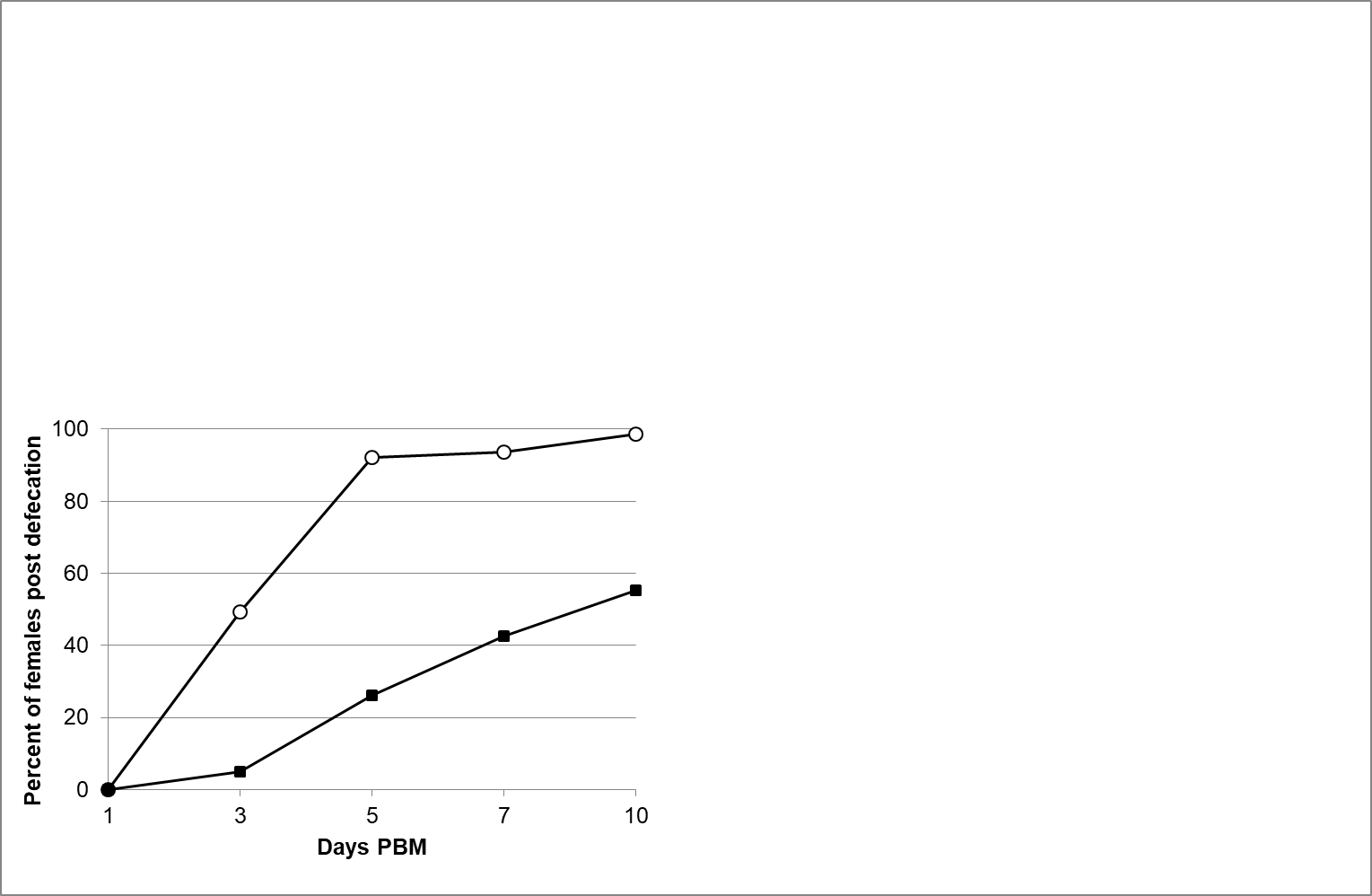


**S2 Figure. Effect of chitinase addition on defecation of *S. schwetzi* females.**

Supplement: S2 Fig — Defecation of sand flies fed on mixture of inactivated rabbit blood mixed 1:1 with supernatant from the culture of B. bassiana containing chitinase (black squares) was compared with defecation on control females fed on the mixture of inactivated rabbit blood mixed 1:1 with medium for B. bassiana instead of the supernatant (open circles). Defecation status was assessed under the light microscope. Numbers of females were: 48, 81, 61, 75 and 96 for chitinase-treated group and 46, 77, 51, 62 and 66 for the control group in days 1, 3, 5, 7 and 10 PBM, respectively. The between–groups differences were significant by days 3–10 PBM (P < 0.05, tested by proportional test with Holm-Bonferroni correction). (DOCX) [file pntd.0006382.s002.docx]

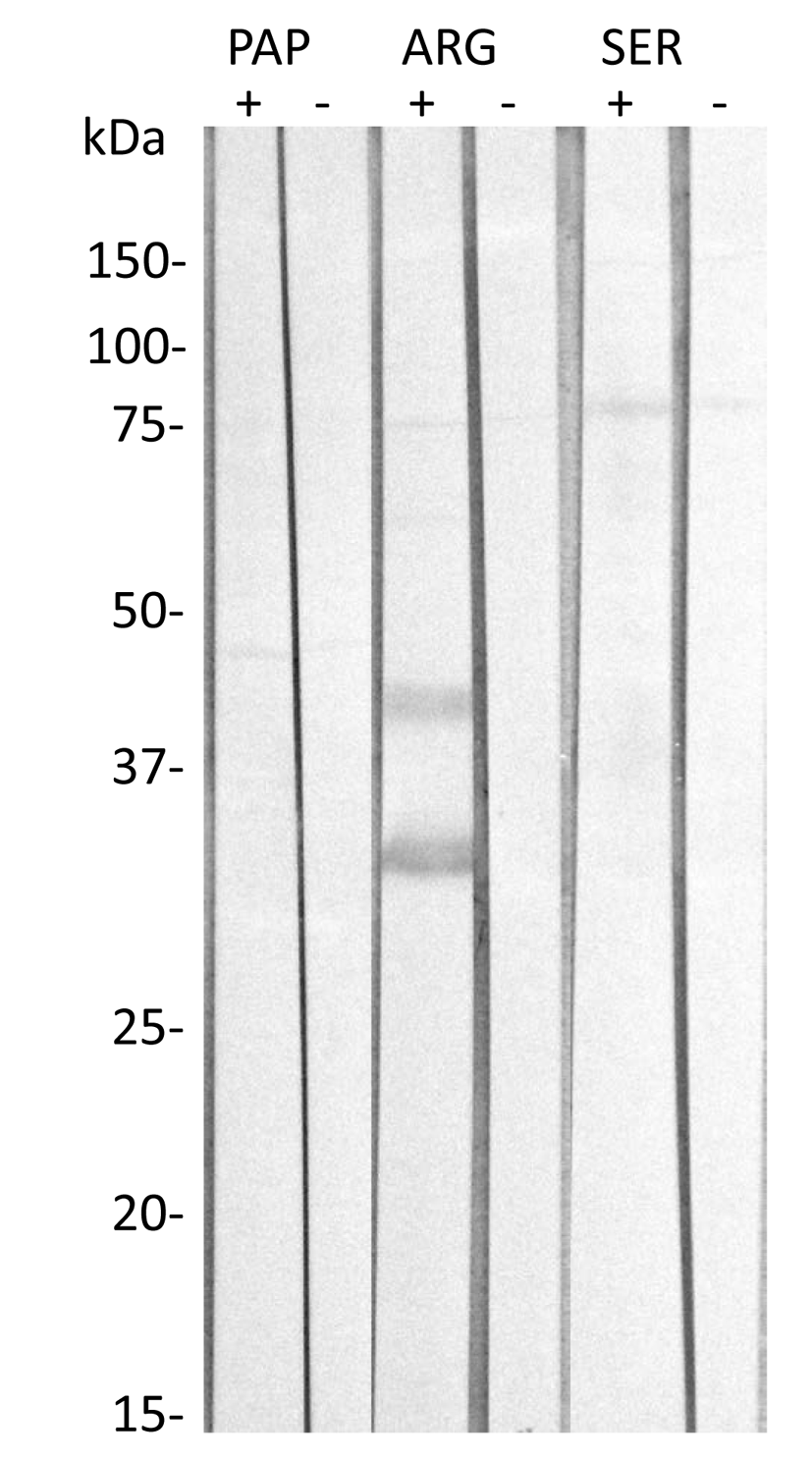

Supplement: S3 Fig — Gut homogenates were fractionated on SDS-PAGE, transferred to nitrocellulose membrane and incubated with biotinylated Helix pomatia lectin (HPA) specifically binding GalNAc. PAP, P. papatasi; ARG, P. argentipes; SER, S. schwetzi; +, separated gut homogenate incubated with lectin HPA; -, incubation of the homogenate with HPA preincubated with specific GalNAc. (TIF) [file pntd.0006382.s003.tif]
