# Supplementary material for: Refractoriness of Sergentomyia schwetzi to Leishmania spp. is mediated by the peritrophic matrix
Source: PLoS Negl Trop Dis. 2018 Apr 4;12(4):e0006382. doi: 10.1371/journal.pntd.0006382 (PMC5902042; doi:10.1371/journal.pntd.0006382)
Supplement: S1 Table — (DOCX) [file pntd.0006382.s004.docx]

Table S1: Effect of chitinases from *S. griseus* and *T. viride* on *Leishmania* growth *in vitro*:

|  | Concentration of chitinase | | | | | | Control  without chitinase |
| --- | --- | --- | --- | --- | --- | --- | --- |
|  | 2.5U/ml | 1.2U/ml | 0.6U/ml | 0.3U/ml | 0.15U/ml | 0.07U/ml |  |
| Medium with chitinase from *S. griseus* | 0 | 0 | 0 | 2 x10^5^/ ml | 1 x10^5^ / ml | 1 x10^5^ / ml | 8-9x10^6^/ml |
| Blood with chitinase from *S. griseus* | 1 x10^5^ / ml | 1 x10^5^ / ml | 1 x10^5^ / ml | 3 x10^5^ / ml | 5 x10^5^ / ml | 2 x10^5^ / ml | 4 x10^5^ / ml |
| Medium with chitinase from *T. viride* | 0 | 0 | 3 x10^5^ / ml | 4 x10^5^ / ml | 8 x10^5^ / ml | 8 x10^5^ / ml | 6-7x10^6^/ml |

Parasites numbers in wells with different concentration of chitinase.
